# Buprenorphine/naloxone induction for treatment of acute on chronic pain using a micro-dosing regimen: A case report

**DOI:** 10.1080/24740527.2019.1599279

**Published:** 2019-04-25

**Authors:** Raman Sandhu, Rebecca Zivanovic, Sukhpreet Klaire, Mohammadali Nikoo, Jennifer Rozylo, Pouya Azar

**Affiliations:** a University of British Columbia, Vancouver, British Columbia, Canada; b British Columbia Centre on Substance Use, Vancouver, British Columbia, Canada; c Addiction and Concurrent Disorders Group, Institute of Mental Health, University of British Columbia, Vancouver, British Columbia, Canada; d Vancouver General Hospital, DHCC, Vancouver, British Columbia, Canada

**Keywords:** chronic pain, opioid, buprenorphine/naloxone, micro-dosing, Bernese method

## Abstract

**Background**: Due to its unique pharmacologic properties, efficacy as an analgesic, and role as a first-line medication for the treatment of opioid use disorder, sublingual buprenorphine has emerged as a treatment for patients with concurrent chronic pain and opioid use disorders. One challenge to utilizing buprenorphine is that precipitated opioid withdrawal can result if this medication is initiated in the presence of other opiates with lesser binding affinities. Micro-dosing induction regimens utilize a slower titration to avoid the need for a period of abstinence from other opiates and decrease the risk of precipitated withdrawal.

**Aims**: The aim of this article is to present a case where a standardized micro-dosing induction regimen was used to transition a patient from other opiate analgesia to a sublingual formulation of buprenorphine/naloxone.

**Methods**: This case took place on an inpatient neurosurgical unit of a Canadian tertiary-care city hospital. Written informed consent was collected prior to a detailed chart review.

**Results**: Here we present a case of a postoperative neurosurgical inpatient who was referred to our team for pain management in the context of chronic pain and a past history of opioid use disorder. She was successfully transitioned to buprenorphine/naloxone, replacing all other opioid analgesia, without a period of opioid withdrawal using a micro-dosing induction regimen.

**Conclusions**: Sublingual buprenorphine/naloxone can be safe and effective for treatment of chronic pain, particularly for those with past or current opioid use disorder. Micro-dosing provides a preferable induction strategy for patients who are not able to tolerate the requirement for moderate opioid withdrawal prior to initiation with existing regimens.

## RÉSUMÉ

**Contexte**: En raison de ses propriétés pharmacologiques uniques, de son efficacité en tant qu’analgésique et de son rôle de médication de première ligne pour le traitement du trouble de l’usage d’opioïdes, la buprénorphine sublinguale s’est imposée comme traitement pour les patients qui souffrent simultanément de douleur chronique et d’un trouble de l’usage d’opioïdes. L’un des défis liés à l’utilisation de la buprénorphine est qu’elle peut donner lieu à un sevrage précipité des opioïdes si l’usage de cette médication est initié en présence d’autres opiacés dont les affinités de liaison sont moindres. Les traitements d’induction par microdosage utilisent un titrage plus lent afin d’éviter qu’une période d’abstinences des autres opiacés soit nécessaire et diminuer le risque de sevrage précipité.

**Objectifs**: Présenter un cas où un traitement d’induction par microdosage a été utilisé pour assurer la transition d’une patiente utilisant d’autres d’autres analgésiques opiacés vers une formulation sublinguale de buprénorphine/naloxone.

**Méthodes**: Ce cas s’est déroulé dans l’unité d’hospitalisation en neurochirurgie d’un hôpital de soins tertiaires d’une ville canadienne. Un consentement éclairé signifié par écrit a été recueilli avant l’examen approfondi des dossiers.

**Résultats**: Nous présentons ici le cas d’une patiente hospitalisée en neurochirurgie qui a été référée à notre équipe après son opération, pour la prise en charge de la douleur dans un contexte de douleur chronique et d’antécédents d’usage d’opioïdes. En ayant recours à un traitement d’induction par microdosage, elle a fait la transition vers la buprénorphine/naloxone et a remplacé tous les autres analgésiques opioïdes sans période de sevrage des opioïdes.

**Conclusions**: La buprénorphine/naloxone sublinguale peut être sécuritaire et efficace pour le traitement de la douleur chronique, particulièrement pour les personnes qui ont un trouble de consommation d’opiacés passé ou actuel. Le microdosage constitue une stratégie d’induction préférable pour les patients qui ne peuvent pas tolérer la nécessité d’un sevrage modéré des opioïdes avant de débuter les traitements existants.

## Introduction

Chronic pain has a prevalence ranging between 11% and 40% in adult populations.^1–3^ Opioid analgesics have been used effectively to rapidly treat acute pain, but the benefits of using these medications for chronic pain are questionable.^4^ There are concerns around safety, dependence, and misuse of opioid analgesics, especially when prescribed in high doses.^5^ Due to its unique pharmacologic properties, efficacy as an analgesic, and role as a first-line medication for the treatment of opioid use disorder, sublingual buprenorphine as a treatment for chronic pain has emerged as a recent area of interest. Though the transdermal and buccal formulations are formally indicated for chronic pain, there is growing evidence for the use of sublingual buprenorphine in the same population.^6–10^ Compared directly to full μ-opioid agonists, treatment of chronic pain with sublingual buprenorphine is associated with decreased pain and improved quality of life.^11^

Buprenorphine is a semisynthetic opioid with high μ-opioid receptor affinity, partial μ-opioid receptor agonism, κ-opioid receptor inverse agonism, δ-opioid receptor antagonism, and slow rate of dissociation from the μ-opioid receptor.^6,9,12^ These characteristics provide buprenorphine a novel mechanism of action for pain relief, including possible reversal of opioid-induced hyperalgesia.^6,9,13^ The pharmacokinetics of buprenorphine result in a favorable safety profile compared to full μ-opioid agonists due to a ceiling effect on respiratory depression and the ability for rapid titration.^6,14^ Buprenorphine is sometimes combined with naloxone in a sublingual formulation to discourage intravenous use.^15^

One challenge to utilizing buprenorphine is that precipitated opioid withdrawal can result if this medication is initiated in the presence of other opiates with lesser binding affinities. Therefore, moderate opioid withdrawal has historically been required prior to initiation to ensure that μ-opioid receptors are unoccupied when buprenorphine is introduced. This can be a significant barrier for patients, especially due to the expectation of significantly increased pain during the withdrawal period.^16,17^

To combat these limitations of conventional buprenorphine inductions, novel approaches to induction, including utilization of short-term transdermal buprenorphine and sublingual micro-dosing, are being explored.^17,18^ Micro-dosing uses a slower titration in small “micro” doses to avoid the need for a period of abstinence and decrease the risk of precipitated withdrawal. These small, incremental doses can be administered with overlapping use of full μ-opioid agonists. A micro-dosing schedule for sublingual buprenorphine was first introduced by a case series of two patients describing successful induction in opioid use disorder.^17^ Both patients tolerated this induction without experiencing precipitated withdrawal or the need for opioid withdrawal symptoms prior to induction. This method was coined “the Bernese method.”^17^ The pharmacological hypothesis tested with the Bernese method is that small amounts of buprenorphine should not precipitate opioid withdrawal and would eventually accumulate at the receptor because of the long half-life; over time, buprenorphine would gradually replace the full μ-agonist at the opioid receptor.

Here we present a case of a postoperative inpatient with pre-existing chronic pain and a history of opioid use disorder who had a successful buprenorphine/naloxone induction, without a period of withdrawal or heightened pain, using a standardized micro-dosing regimen.

## Materials and methods

This is a case study of a single patient admitted to a tertiary-care hospital in Vancouver, British Columbia, Canada. Informed consent was collected in writing from the patient prior to data collection. The paper chart and online medical records from the hospital stay were reviewed and summarized.

## Results

The patient was a 59-year-old woman of First Nations ancestry living with her son in a rural town in British Columbia, Canada. She was divorced and supported by social disability. She had been transferred urgently from her local hospital to our hospital for emergent management of an acute subarachnoid hemorrhage secondary to a ruptured aneurysm.

Her past medical history was significant for 12 years of chronic pain with contributing diagnoses of osteoarthritis, migraine headache, fibromyalgia, and mild peripheral neuropathy. She also had hepatitis C virus, major depressive disorder, and mild cognitive impairment. Her medications prior to hospitalization were as follows:

1. Oxycodone/acetaminophen 10 mg/650 mg orally three times a day
2. Amitriptyline 50 mg orally once daily
3. Duloxetine 30 mg orally twice daily
4. Baclofen 10 mg orally three times a day as needed
5. Rabeprazole 20 mg orally once daily.

She had been using oxycodone for pain relief for many years. She reported taking duloxetine and amitriptyline primarily for treatment of depression.

She had a 25-year history of intravenous heroin use with last use occurring 5 years prior to hospitalization. She had never received opioid agonist therapy and had never been to a treatment facility. She had no history of overdoses. Concern regarding past prescription oxycodone overuse was shared with the care team by her son. There was no history of buying opiate analgesia from the street or diversion. She had a 45 pack-year history of cigarette smoking, having cut down from one pack per day to half a pack per day in the last year. She denied using alcohol or other illicit substances.

Her subarachnoid hemorrhage was managed surgically by neurosurgery. Postoperatively she had a communicating hydrocephalus, which was treated with external ventricular drain and then a lumbar drain. Eventually her hydrocephalus stabilized and lumbar drain was removed.

Postoperatively our complex pain and addiction consult service became involved for pain management. This service is staffed by psychiatrists with subspecialty training in pain and addictions who provide consultations for inpatients on medical, surgical, and psychiatric inpatient wards. Their scope includes psychological and pharmacological management of pain and substance use disorders. She described ongoing headache and stabbing neck pain radiating to her neck and shoulders. She had minimal pain relief despite being treated with

1. Acetaminophen 650 mg orally four times a day
2. Oxycodone 10 mg orally three times a day
3. Hydromorphone 0.4–2 mg orally every 2 h as needed
4. Hydromorphone 0.1–0.4 mg intravenous every hour as needed.

She was using approximately 5 mg of hydromorphone intravenous as needed daily. Her home medications were continued and she was also treated with nimodipine for prevention of cerebral vasospasm.

Considering the patient’s history of illicit heroin use and current use of high-dose opioid analgesics for chronic pain, a safer opioid medication was recommended and the patient agreed to this. Buprenorphine/naloxone micro-dosing was initiated on postoperative day 4 at 0.25 mg sublingual twice a day with a daily titration to achieve a dose of 12 mg sublingual daily in one week. The full titration schedule is detailed in Table 1. The prescribed intravenous hydromorphone was discontinued on day 1 of buprenorphine/naloxone induction but other opioid analgesia (oxycodone 10 mg orally three times a day and hydromorphone 0.4–2 mg orally every 2 h as needed) were continued during the micro-dosing regimen. Oral oxycodone and hydromorphone were discontinued on day 7 of the induction.

**Table 1.** Buprenorphine/naloxone micro-dosing titration schedule.^a^

Day	Buprenorphine dose	Buprenorphine/naloxone strength to use
1	0.25 mg sublingual daily	Buprenorphine 2 mg/naloxone 0.5 mg
2	0.25 mg sublingual twice daily	Buprenorphine 2 mg/naloxone 0.5 mg
3	0.5 mg sublanguage twice daily	Buprenorphine 2 mg/naloxone 0.5 mg
4	1 mg sublingual twice daily	Buprenorphine 2 mg/naloxone 0.5 mg
5	2 mg sublingual twice daily	Buprenorphine 2 mg/naloxone 0.5 mg
6	4 mg sublingual twice daily	Buprenorphine 2 mg/naloxone 0.5 mg
7	12 mg sublingual daily	Buprenorphine 2 mg/naloxone 0.5 mg

^a^Starting on day 8, continue buprenorphine/naloxone 12 mg/3 mg (one tab) sublingual once daily.

Within 2 days of initiation of buprenorphine/naloxone micro-dosing, she described moderate pain relief. Throughout the titration, she denied cravings for opioids and did not experience opioid withdrawal symptoms. All other opioid analgesics were discontinued on day 7 of the buprenorphine/naloxone micro-dosing schedule and she was continued on buprenorphine/naloxone 12 mg daily alone. At this point she described full relief of head and neck pain. She tolerated buprenorphine/naloxone well throughout the micro-dosing schedule and reported no adverse effects.

## Discussion

Here we presented a case of a postoperative inpatient with ongoing acute on chronic pain complicated by a history of opioid use disorder who was referred to our team for inadequate pain control despite high-dose opioid analgesia. She tolerated a buprenorphine/naloxone induction using a micro-dosing schedule without symptoms of precipitated withdrawal or worsened pain. This allowed for induction without the need for preceding opioid withdrawal symptoms. Given the long-term use of high-dose opioids, this patient would have likely experienced significant opioid withdrawal symptoms as well as uncontrolled pain if a traditional buprenorphine induction had been employed. This protocol allowed for simplification of the patient’s analgesic regimen in the postoperative period and resulted in her being discharged home on a safer opioid analgesic for long-term treatment of her chronic pain compared to what she had been prescribed prior to hospitalization. The benefits here include significantly reduced risk of opioid tolerance, dependence, misuse, and overdose.

The previously published case series on micro-dosing utilized sublingual buprenorphine without naloxone and was treating patients with opioid use disorder in the outpatient setting. In contrast to our study, the opioids used by those patients included street heroin and prescribed forms of opioid agonist therapy including diacetylmorphine and methadone.^17^ These two patients stabilized on buprenorphine 12 mg and 24 mg sublingual daily, respectively.^17^ Our patient did not have active illicit opioid use nor was she on opioid agonist therapy. She was, however, taking a considerable amount of opioid analgesia, including oxycodone (oral) and hydromorphone (oral and intravenous). She similarly stabilized on a dose of suboxone/naloxone of 12 mg sublingual daily. The inpatient setting of our study allowed for regular monitoring for complications.

A recent review of various formulations of buprenorphine for treatment of chronic pain found that the majority of 25 studies reviewed showed a significant decrease in pain versus comparator, which was generally placebo and more rarely other analgesics.^9^ The vast majority of the studies examined transdermal buprenorphine with some finding that compared to tramadol and immediate-release oxycodone, transdermal buprenorphine was actually found to be inferior.^19,20^ A single study included in this review compared buprenorphine/naloxone once daily dosing (average dose 14.93/3.73 mg) versus methadone for treatment of chronic pain in patients with opioid use disorder and found a significant reduction in pain in both groups.^21^ An earlier review of sublingual buprenorphine as an analgesic in chronic pain concluded, based on ten studies that were mostly observational in nature, that there was a plausible role for sublingual buprenorphine in chronic pain but that further high-quality studies were needed.^7^ Another review similarly provides support for transdermal and buccal formulations of buprenorphine in chronic pain commenting on the general underutilization of this effective medication and frequency of underdosing due to gaps in practitioner education.^10^

The case presented here represents not only a novel induction method but also a novel application given that the patient was experiencing acute on chronic pain. This adds to existing evidence that buprenorphine has safety and efficacy equivalent to that of morphine when used in inpatient settings to treat acute pain with the added benefit of less frequent administration.^22^ Interestingly, there is disagreement in the literature on treatment of acute pain in the perioperative period for patients who are on buprenorphine prior to surgery, with some studies suggesting that buprenorphine may result in difficult-to-control postoperative pain, leading some to suggest that it should be discontinued prior to elective surgeries.^23^ However, our case suggests that buprenorphine/naloxone, used mindfully, may be a useful tool rather than a barrier to treatment in the postoperative period. Additionally, a micro-dosing regimen of buprenorphine may be useful in such cases where buprenorphine needs to be restarted after being discontinued for perioperative pain management.

Currently, sublingual buprenorphine, including the buprenorphine/naloxone formulation, is considered off-label for use in chronic pain, in contrast to the buprenorphine transdermal patch and buccal film.^10^ Growing evidence for the efficacy and safety of sublingual buprenorphine in chronic pain should prompt re-evaluation of this. We specifically want to highlight the need for future research into the efficacy of sublingual buprenorphine/naloxone for chronic pain given its superior safety profile in the context of the opioid overdose crisis. Future research is needed to evaluate the efficacy and safety for micro-dosing regimens for buprenorphine/naloxone induction in larger populations of patients with chronic pain in both the inpatient and outpatient settings. Careful attention should be paid to the potential for buprenorphine/naloxone to replace less-safe alternatives such as other opioid analgesics, including methadone. Future research should also look beyond safety and efficacy into the impact that transition to once-daily administration of buprenorphine/naloxone for pain control in complex patients can have on quality of life. In our case, we show how a complex analgesia regimen requiring many daily doses can be effectively consolidated into a single-dose treatment that manages pain consistently over a 24-h period.

In conclusion, this case represents the first documentation in the literature of a micro-dosing regimen being used for transition from other opioids to buprenorphine/naloxone for treatment of chronic pain. It provides important preliminary evidence that sublingual buprenorphine/naloxone can be safe and effective for treatment of chronic pain without the requirement for opioid withdrawal prior to initiation.

## References

[1] Birse TM, Lander J. Prevalence of chronic pain. Can J Public Health. 1998;89:129–31. 9583256 10.1007/BF03404405 PMC6990192

[2] Breivik H, Collett B, Ventafridda V, Cohen R, Gallacher D. Survey of chronic pain in Europe: prevalence, impact on daily life, and treatment. Eur J Pain. 2006;10(4):287–333. doi:10.1016/j.ejpain.2005.06.009. 16095934

[3] Schopflocher D, Taenzer P, Jovey R. The prevalence of chronic pain in Canada. Pain Res Manag. 2011;16:445–50. 22184555 10.1155/2011/876306 PMC3298051

[4] Dowell D, Haegerich TM, Chou R. CDC guideline for prescribing opioids for chronic pain–United States, 2016. JAMA. 2016;315(15):1624–45. doi:10.1001/jama.2016.1464. 26977696 PMC6390846

[5] Volkow ND, McLellan AT. Opioid abuse in chronic pain–misconceptions and mitigation strategies. N Engl J Med. 2016;374(13):1253–63. doi:10.1056/NEJMra1507771. 27028915

[6] Chen KY, Chen L, Mao J. Buprenorphine-naloxone therapy in pain management. Anesthesiology. 2014;120(5):1262–74. doi:10.1097/ALN.0000000000000170. 24509068 PMC3999180

[7] Cote J, Montgomery L. Sublingual buprenorphine as an analgesic in chronic pain: a systematic review. Pain Med. 2014;15(7):1171–78. doi:10.1111/pme.2014.15.issue-7. 24995716

[8] British Columbia Centre on Substance Use. A guideline for the clinical management of opioid use disorder. Victoria, British Columbia: British Columbia Ministry of Health; 2017.

[9] Aiyer R, Gulati A, Gungor S, Bhatia A, Mehta N. Treatment of chronic pain with various buprenorphine formulations: a systematic review of clinical studies. Anesth Analg. 2018;127(2):529–38. doi:10.1213/ANE.0000000000003570. 29239947

[10] Fishman MA, Kim PS. Buprenorphine for chronic pain: a systematic review. Current Pain and Headache Reports. 2018;22(83):1–7. doi:10.1007/s11916-018-0732-2. 30291571

[11] Daitch D, Daitch J, Novinson D, Frey M, Mitnick C, Pergolizzi J. Conversion from high-dose full-opioid agonists to sublingual buprenorphine reduces pain scores and improves quality of life for chronic pain patients. Pain Med. 2014;15(12):2087–94. doi:10.1111/pme.12520. 25220043

[12] Davis MP, Pasternak G, Behm B. Treating chronic pain: an overview of clinical studies centered on the buprenorphine option. Drugs. 2018;78(12):1211–28. doi:10.1007/s40265-018-0953-z. 30051169 PMC6822392

[13] Lutfy K, Eitan S, Bryant CD, Yang YC, Saliminejad N, Walwyn W, Kieffer BL, Takeshima H, Carroll FI, Maidment NT, et al. Buprenorphine-induced antinociception is mediated by mu-opioid receptors and compromised by concomitant activation of opioid receptor-like receptors. J Neurosci. 2003;23(32):10331–37. doi:10.1523/JNEUROSCI.23-32-10331.2003. 14614092 PMC6741014

[14] Walsh SL, Preston KL, Stitzer ML, Cone EJ, Bigelow GE. Clinical pharmacology of buprenorphine: ceiling effects at high doses. Clin Pharmacol Ther. 1994;55(5):569–80. doi:10.1038/clpt.1994.71. 8181201

[15] Chiang CN, Hawks RL. Pharmacokinetics of the combination tablet of buprenorphine and naloxone. Drug Alcohol Depend. 2003;70(2 Suppl):S39–47. doi:10.1016/S0376-8716(03)00058-9. 12738349

[16] Daniulaityte R, Carlson R, Brigham G, Cameron D, Sheth A. “Sub is a weird drug:” A web-based study of lay attitudes about use of buprenorphine to self-treat opioid withdrawal symptoms. Am J Addict. 2015;24(5):403–09. doi:10.1111/ajad.12300. 26009867 PMC4527156

[17] Hämmig R, Kemter A, Strasser J, von Bardeleben U, Gugger B, Walter M, Dürsteler KM, Vogel M. Use of microdoses for induction of buprenorphine treatment with overlapping full opioid agonist use: the Bernese method. Subst Abuse Rehabil. 2016;7:99–105. doi:10.2147/SAR.S109919. 27499655 PMC4959756

[18] Kornfeld H, Reetz H. Transdermal buprenorphine, opioid rotation to sublingual buprenorphine, and the avoidance of precipitated withdrawal: a review of the literature and demonstration in three chronic pain patients treated with butrans. Am J Ther. 2015;22(3):199–205. doi:10.1097/MJT.0b013e31828bfb6e. 23846520

[19] Poulain P, Denier W, Douma J, Hoerauf K, Samija M, Sopata M, Wolfram G. Efficacy and safety of transdermal buprenorphine: a randomized, placebo-controlled trial in 289 patients with severe cancer pain. J Pain Symptom Manage. 2008;36(2):117–25. doi:10.1016/j.jpainsymman.2007.09.011. 18411010

[20] Gordon A, Callaghan D, Spink D, Cloutier C, Dzongowski P, O‘Mahony W, Sinclair D, Rashiq S, Buckley N, Cohen G, et al. Buprenorphine transdermal system in adults with chronic low back pain: a randomized, double-blind, placebo-controlled crossover study, followed by an open-label extension phase. Clin Ther. 2010;32(5):844–60. doi:10.1016/j.clinthera.2010.06.017. 20685494

[21] Neumann AM, Blondell RD, Jaanimägi U, Giambrone AK, Homish GG, Lozano JR, Kowalik U, Azadfard M. A preliminary study comparing methadone and buprenorphine in patients with chronic pain and coexistent opioid addiction. J Addict Dis. 2013;32(1):68–78. doi:10.1080/10550887.2012.759872. 23480249 PMC3604999

[22] White LD, Hodge A, Vlok R, Hurtado G, Eastern K, Melhuish TM. Efficacy and adverse effects of buprenorphine in acute pain management: systematic review and meta-analysis of randomized controlled trials. Br J Anaesth. 2018;120(4):668–78. doi:10.1016/j.bja.2017.11.086. 29576108

[23] Anderson T, Quaye A, Ward E, Wilens T, Hillard P. To stop or not, that is the question: acute pain management for the patient on chronic buprenorphine. Anesthesiology. 2017;125:1180–86. doi:10.1097/ALN.0000000000001633. PMC7041233 28511196

